# Aptamer-modified gold nanoparticles for rapid aggregation-based detection of inflammation: an optical assay for interleukin-6

**DOI:** 10.1007/s00604-019-3975-7

**Published:** 2019-12-04

**Authors:** Susan Giorgi-Coll, María J. Marín, Olajumoke Sule, Peter J. Hutchinson, Keri L.H. Carpenter

**Affiliations:** 1 Department of Clinical Neurosciences, Division of Neurosurgery, University of Cambridge, Box 167, Cambridge Biomedical Campus, Cambridge, CB2 0QQ UK; 2 School of Chemistry, University of East Anglia, Norwich Research Park, Norwich, NR4 7TJ UK; 3 Clinical Microbiology and Public Health Laboratory, Cambridge University Hospitals NHS Trust, Box 236, Addenbrooke’s Hospital, Cambridge, CB2 0QQ UK; 4 Department of Clinical Neurosciences, Wolfson Brain Imaging Centre, University of Cambridge, Box 65, Cambridge Biomedical Campus, Cambridge, CB2 0QQ UK

**Keywords:** Cytokines, Colorimetry, Metal nanoparticles, Sepsis, Diagnostics, Point-of-care assay

## Abstract

A proof-of-concept aptamer-based optical assay is described for the determination of the immuno signalling molecule interleukin-6 (IL-6), a key marker of acute inflammation. The optical assay is based on the aggregation of gold nanoparticles (AuNP) coated in two complimentary “sandwich-style” aptamers, each with different IL-6 target moieties. IL-6 will recognise the complimentary aptamer pair and bind to it, thereby causing the aggregation of the corresponding functionalised nanoparticles. The aggregation of the AuNPs after exposure to IL-6 induces a visible colour change from red to pink, with a corresponding change in the absorption maximum from 520 to 540 nm. The change in the absorption maximum can be monitored visually, or by using a spectrophotometer or a plate reader. The optimal size and functionalisation of aptamer-coated AuNPs, and the potential assay formats were investigated using UV-vis spectrophotometry, transmission electron microscopy, and dynamic light scattering. The optical assay was applied for detecting mouse IL-6 in a mixed protein solution as a representative biological sample. The assay works in the 3.3 to 125 μg·mL^−1^ IL-6 concentration range, and the detection limit (at S/*N* = 3) is 1.95 μg·mL^−1^. This study was performed as a proof-of-concept demonstration of this versatile assay design, with a view to developing a similar assay for use in clinical samples in future.

**Electronic supplementary material:**

The online version of this article (10.1007/s00604-019-3975-7) contains supplementary material, which is available to authorized users.

**Graphical abstract:**
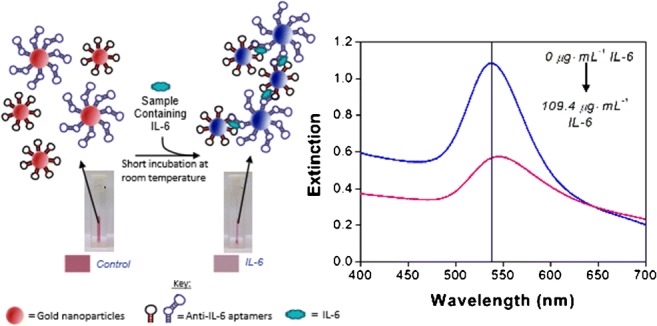
Schematic representation of the aggregation of aptamer-functionalised nanoparticles in the presence of interleukin-6 (IL-6). The presence of mouse IL-6 in a mixed protein solution leads to a visible colour change, and a change in the absorption spectrum of the nanoparticles.

## Introduction

Inflammation, the body’s immune response to infection or injury, plays a key role in almost all pathological conditions. Inflammation is mediated by immune molecules which act as chemical messengers yielding a response from other cells. Different types of immune molecules have been associated with driving inflammation, or a redirection to a healing function. There is increasing evidence that early detection of inflammatory molecules can enable timely clinical intervention with potentially life-saving consequences for patients. This is particularly true for pathological conditions in hidden environments such as the brain, where changes in inflammatory processes are not always immediately obvious from a clinical perspective.

Interleukin-6 (IL-6) is an immune molecule associated with acute inflammation, particularly in response to recent infection or injury [1]. High levels of IL-6 measured in cerebrospinal fluid (CSF) and plasma have been associated with pathological inflammation leading to poor clinical outcome in patients with brain injuries, such as traumatic brain injury (TBI) [2, 3] and subarachnoid haemorrhage (SAH) [4, 5]. IL-6 above a certain threshold in CSF drain samples has been recognised as a potentially important diagnostic marker of bacterial meningitis [6]. Bedside detection of raised IL-6 would potentially enable rapid treatment of meningitis with anti-inflammatory steroids, thereby decreasing the risk of mortality [6, 7]. IL-6 can also be used as an early indicator of potentially life-threatening infection. Sepsis, which is systemic, uncontrolled inflammation caused by the body’s reaction to infection, is one of the most common causes of deaths in a hospital environment, and is especially common in the elderly [8]. Conventional diagnosis of sepsis relies on recognition of infection, which can be challenging in comatose patients. Positive microbiological cultures (which can take days) remains the gold standard for diagnosis, along with key clinical assessments including temperature, white cell count (WCC), C-reactive protein (CRP), and blood lactate [9, 10]. Significant developments in the field of sepsis diagnosis attempt to address the lengthy time required to perform blood cultures. These include new real-time PCR, multiplex PCR-based assays, and microarray techniques for pathogen identification directly from blood. Although these new methods enable more rapid diagnosis, current limitations include high cost (£140–£160/test) and specialised laboratory equipment requirements [11]. Bedside detection of raised IL-6 levels in the blood would aid the diagnosis of infection, thereby enabling the fast and effective treatment necessary to combat this disease [8, 10, 12].

The growing trend for transition of diagnostic tests from the laboratory to the point-of-care reflects the mounting evidence that early detection of changes in the body enable rapid clinical intervention with maximum benefit. This is particularly relevant in cases of increased inflammation. Current commercially available technology employs antibodies as detection agents, in both the clinical laboratory, and in point-of-care diagnostics. An example is a bedside interleukin-6 test for the early detection of infection in expectant mothers, to prevent pre-term births [13]. This test uses antibodies to bind IL-6 and enable detection. Antibodies are used as the recognition unit in the majority of methods reported in the literature that could be potentially apply for the detection of IL-6 in clinical diagnosis (Table S1). Antibodies are mass-produced in animals, hybridoma cell ines or genetically-modified bacteria, with the inherent risk of inter-batch variability; they require refrigerated storage and specific conditions for optimal performance, and they can degrade with time, giving them limited shelf-life. These disadvantages can limit the practicality of antibody-based tests, and seeking alternatives to antibodies is therefore of tremendous importance. Active research in this area has led to the development of an automated system for the synthetic production of aptamers, short DNA strands. Aptamers are tuneable and enable high-affinity binding, resulting in improved assay sensitivity [14, 15]. Crucially, their synthetic method of production means that they are cheaper to produce, and chemically identical than antibodies. Aptamers are also more stable than antibodies and can be stored for long periods without refrigeration [16]. The use of aptamers in place of antibodies can potentially improve test sensitivity over current state-of-the-art bedside tests, and enable smaller sample volumes to be analysed.

The physical properties of gold nanoparticles (AuNP) cause them to be brightly coloured in solution, and this colour visibly changes in response to AuNP aggregation. Diagnostic tests based on the aggregation of AuNP have been demonstrated as highly effective for the detection of toxins, bacteria, viruses (in allantoic fluid), and immune molecules in human biological samples [17–19]. Tests of this nature offer significant advantages over the current state-of-the-art in terms of sensitivity, reliability, cost, and easy storage without refrigeration. They are particularly practical in a bedside setting as they can be performed quickly and easily (in 20 min or less) at room temperature and without sample pre-treatment [20]. AuNPs functionalised with heparin have been recently reported for enhanced microdialysis sampling of cytokines [21].

**Fig. 1:**
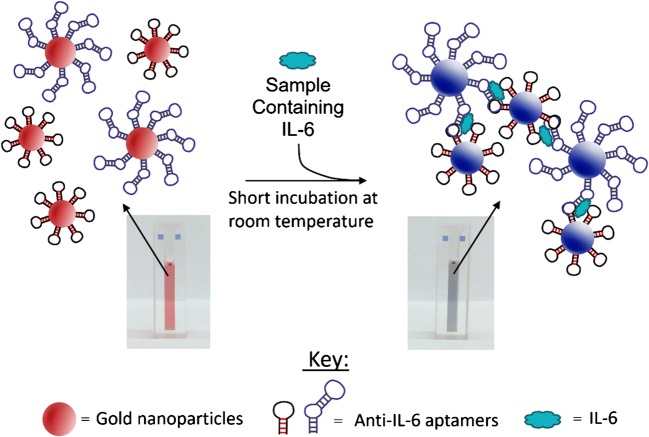
A schematic of the aptamer-gold nanoparticle-based aggregation assay for the detection of mouse IL-6

In the present study, we report the development and preliminary trial of a proof-of-concept version of a novel bedside test for the detection of IL-6 in biological samples from patients with brain injury or infection. The test is based on AuNP functionalised with a ‘sandwich-pair’ of aptamers specific for murine IL-6. To the best of our knowledge, this is the first time that a ‘sandwich-pair’ of aptamers is reported for the optical detection of IL-6 using gold nanoparticles. The presence of IL-6, which binds to multiple aptamers on the AuNP surface, results in aggregation of the AuNP as schematically shown in Fig. 1. In this pilot study, the optical assay was applied for the detection of mouse IL-6 in a mixed protein solution as a representative biological sample matrix as an initial proof-of-concept. With further development, the new technique may be applicable for the rapid and straightforward detection of acute inflammation in either a mouse model, or in mouse-derived cell culture supernatant. The test is performed without previous sample treatment and requires only 5 min to complete, making it useful in a wide range of clinical settings. The assay design is versatile. In future, it may be adapted to a ‘dip-stick’ format.

## Experimental

### Materials and equipment

High-purity deionised water (dH_2_O) used was of HPLC-grade (18.2 MΩ·cm, Millipore Direct Q5 UV water purification system with LC-Pak polisher, https://www.merckmillipore.com). All reagents were of analytical grade, purchased from Sigma-Aldrich (Poole, Dorset, UK, https://www.sigmaaldrich.com) and used as received unless otherwise stated. Millipore Millex-HV PVDF syringe filter units (0.45 μm) and Millipore Amicon Ultra 0.5 mL centrifuge units (100 k MWCO) were also purchased from Sigma-Aldrich (Poole, Dorset, UK, https://www.sigmaaldrich.com). Sodium phosphate dibasic, sodium chloride, sodium dodecyl sulfate (SDS), and potassium chloride were purchased from BDH Laboratory Supplies (Poole, UK, https://www.coleparmer.co.uk). 1.5 mL and 5 mL Lo-bind Eppendorf tubes were purchased from Thermo Fisher (Loughborough, UK, https://www.thermofisher.com). Recombinant murine IL-6 standard was purchased from PeproTech (London, UK, https://www.peprotech.com). Complementary sandwich pair anti-murine IL-6 aptamers (ATW0082 and ATW0077, with terminal thiol-modification) and buffers (resuspension buffer and aptamer reducing buffer) were purchased from BasePair Biotechnologies Inc. (Pearland, Texas, USA, https://www.basepairbio.com). Holey carbon film 300 mesh copper grids were purchased from Agar Scientific (Stansted, UK, http://www.agarscientific.com). ProcartaPlex human cytokine and chemokine standard mixes A and B, and Universal Assay Buffer were purchased from Affymetrix eBioscience (Hatfield, UK, https://www.thermofisher.com).

All centrifugation steps were performed using a Hettich 320R centrifuge. Gentle agitation was achieved using a Grant-Bio PS-3D Sunflower mini-shaker. Citrate AuNP (cAuNP) synthesis was performed using a VWR hotplate magnetic stirrer with temperature probe. UV-Vis absorption spectra (200–800 nm) were recorded on a Hitachi U-3000 spectrophotometer at room temperature. Quartz cuvettes with a 1 cm path length were used. Transmission electron microscope (TEM) images were obtained using a FEI Tecnai F20 FEGTEM operating at 200 kV. Dynamic light scattering (DLS) measurements of the 15 nm cAuNP were performed on a Malvern Zetasizer Nano ZSP (running on Zetasizer 7.12 software). Measurement time, attenuation factor and detector position were set as automatic. All other DLS measurements were performed using a Wyatt Technologies DynoPro (DLS) Plate Reader II with Corning 96-well plastic-bottom plates (provided by Wyatt Technologies).

### Gold nanoparticle (AuNP) synthesis and characterisation

Citrate-stabilised gold nanoparticles (cAuNP) of ca. 15 nm were synthesised according to the method of Turkevich et al. [22]. Experimental details are given in the Electronic Supporting Material.

Synthesis of ca. 50 nm cAuNP was performed using the seeded growth method (for AuNPs of up to 180 nm) of Bastús et al. [23]*.* Experimental details are given in the Electronic Supporting Material.

### Preparation of aptamer-functionalised AuNP

15 and 50 nm cAuNP were functionalised with thiolated aptamers using the pH-assisted and surfactant-free method previously reported by Zhang et al. [24, 25]. The thiolated aptamers were first resuspended, folded, and reduced in accordance with the manufacturers’ instructions. Briefly, the lyophilised aptamers (as received from the manufacturer) were each reconstituted in ‘resuspension buffer’ to 100 μM. The aptamers were diluted to 1:2 (50 μM) in phosphate-buffered saline (PBS) (10 mM phosphate buffer, 2.7 mM potassium chloride, 137 mM sodium chloride, pH 7.4; with 1 mM MgCl_2_). Folding was performed by heating the aptamers to 95 °C for 5 min, before cooling slowly at room temperature for 15 min. A 1:1 volume ratio of ‘aptamer reducing buffer’ was added to each aptamer solution, followed by a 10 min incubation at room temperature. The aptamers were then diluted to a working concentration (either 22 or 28 μM) in trisodium citrate (10 mM, pH 3.0) for addition to the cAuNP.

Aptamer-functionalised ca. 15 nm AuNP were prepared by adding the aptamers (31.5 μL of a 22 μM solution, prepared as described previously) to an aliquot of 15 nm cAuNP (1.5 mL) in a 1.5 mL Eppendorf tube. For AuNP functionalised with both aptamers on the same particle, the stock solutions of each aptamer (prepared as described above) were mixed (using a 1:1 ratio) prior to addition to the cAuNP. After addition of the aptamers, the solution was then pipette-mixed and left standing for 1 min at room temperature. Trisodium citrate (30 μL of a 500 μM solution, pH 3.0) was added to the AuNP and pipette-mixed, followed by a 10 min incubation at room temperature. NaCl (52.5 μL of a 2 M solution) was then slowly added (drop-wise). The solution was then mixed by pipette and incubated for 20 min on a 3D shaker (60 rpm) at room temperature. The solution, red in colour, was treated with more NaCl (150 μL of a 2 M solution, added dropwise). The solution was then mixed by pipette once again, and incubated for 40 min on a 3D shaker (60 rpm) at room temperature. The aptamer-AuNP were then washed three times to remove any unconjugated aptamers. The solution was centrifuged at 8000 rpm for 30 min in 100 k MWCO ultrafiltration tubes. After the first centrifugation step, the aptamer-AuNP were resuspended in 1.5 mL PB (10 mM, pH 7.4) with 0.05% *w*/*v* tween-20. After the remaining centrifugation steps (and for storage), the aptamer-AuNP were resuspended in 1.5 mL PBS (10 mM phosphate buffer, 2.7 mM potassium chloride, 137 mM sodium chloride, pH 7.4, 1 mM MgCl_2_). Modification of the AuNP surface was determined by UV-Vis spectrophotometry at ca. 520 nm. The ca. 15 nm aptamer-AuNP were then stored at 4 °C before use.

Aptamer-functionalised ca. 50 nm AuNP were prepared by adding the aptamers (180 μL of a 28 μM solution, prepared as described previously) to an aliquot of 50 nm cAuNP (2 mL) in a 5 mL Lo-bind Eppendorf tube. For AuNP functionalised with both aptamers on the same particle, the stock solutions of each aptamer (prepared as described above) were mixed (using a 1:1 ratio) prior to addition to the cAuNP. After addition of the aptamers, the solution was then pipette-mixed and left standing for 1 min at room temperature. Trisodium citrate (100 μL of a 500 μM solution, pH 3.0) was added to the AuNP and pipette-mixed, followed by a 10 min incubation at room temperature. NaCl (70 μL of a 2 M solution) was then slowly added (drop-wise). The solution was then mixed by pipette and incubated for 20 min on a 3D shaker (60 rpm) at room temperature. The solution, red in colour, was treated with more NaCl (200 μL of a 2 M solution, added drop-wise). The solution was then mixed by pipette once again, and incubated for 40 min on a 3D shaker (60 rpm) at room temperature. The aptamer-AuNP were then washed three times to remove any unconjugated aptamers. The solution was then aliquoted into two 2 mL Eppendorf tubes, and centrifuged at 4000 rpm for 15 min to form a pellet. After the first centrifugation step, the aptamer-AuNP were resuspended in 2 mL PB (10 mM, pH 7.4) with 0.05% *w*/*v* Tween-20. After the remaining centrifugation steps (and for storage), the aptamer-AuNP were resuspended in 2 mL 1X PBS buffer (pH 7.4, 1 mM MgCl_2_). Modification of the AuNP surface was determined by UV-Vis spectrophotometry at ca. 540 nm. The ca. 50 nm aptamer-AuNP were then stored at 4 °C before use.

### IL-6 aggregation assays and characterisation by dynamic light scattering (DLS)

Aggregation assays were performed by adding increasing concentrations of IL-6 (step-wise, from 0 to ≤125 μg·mL^−1^) into a sample of aptamer-AuNP (300 μL) in a cuvette. For single-aptamer-AuNP, equal volumes of aptamer-AuNP 1 and aptamer-AuNP 2 were first combined in a cuvette and mixed prior to IL-6 titration. For mixed-aptamer-AuNP the solution was used as prepared. A UV-Vis spectrum was measured before the addition of the IL-6, and 5 min after each subsequent addition. The 5 min incubation prior to each measurement was performed by capping the cuvette and placing it on a 3D shaker (60 rpm) at room temperature. In order to control for the dilution effect, a buffer control was performed alongside the IL-6 test, in which equal volumes of the diluent (without IL-6) were added prior to measurement.

Once the assay had been successfully applied for the measurement of IL-6 in buffer, the aggregation assays were repeated on IL-6 in a representative biological matrix. All conditions of the experiments were performed using the same protocol as for the titration of IL-6 in buffer (as specified above), including the 5 min incubation period at room temperature with light agitation prior to measuring. This matrix comprised of a mixed protein solution containing 0.5 mg·mL^−1^ human serum albumin (HSA) and 34 human cytokines a chemokines, was prepared as follows. The ProcartaPlex mixed human cytokine/chemokine standards (A and B), received as lyophilised powders, were resuspended in accordance with the manufacturers’ instructions. Subsequently, the standards were diluted to 1:100 in ProcartaPlex Universal Assay Buffer with 0.5 mg·mL^−1^ HSA. The cytokine and chemokine concentrations of this matrix solution are as shown in the Electronic Supporting Material (Table S2). For the test, murine IL-6 standard (as a 1 mg·mL^−1^ stock solution in dH_2_O) was added to the matrix prior to titration. For the negative control, an equal volume of dH_2_O (without murine IL-6) was added to the matrix solution prior to testing. A buffer control, consisting of ProcartaPlex Universal Assay Buffer with 0.5 mg·mL^−1^ HSA (without additional proteins), was also tested to check for cross-reactivity with the human proteins.

Upon completion of the aggregation assays, aptamer-AuNP and aggregation assay particle size estimations were performed by DLS using a DynoPro (DLS) Plate Reader II (as previously described).

## Results and discussion

### AuNP synthesis and characterisation

Citrate-capped gold nanoparticles (cAuNPs) were synthesised according to the citrate reduction methods of Turkevich et al. (ca. 15 nm) and Bastús et al. (ca. 50 nm), as described in the Electronic Supporting Material [22, 23]. The average diameters of the cAuNP, measured from TEM images, are 15.0 ± 1.3 nm and 53.0 ± 5.2 nm (*n* = 100) (Fig. S1).

The cAuNP were functionalised with thiolated aptamers via the pH-assisted method of Zhang et al. as described in the Electronic Supporting Material [24, 25]. The binding of the aptamers to the surface of the AuNP is demonstrated by a shift in the absorption wavelength of approximately 3–4 nm following functionalisation. The UV-Vis absorption spectra that support this statement are shown in Fig. S1c and f for 15 nm and 50 nm AuNPs, respectively. This shift to the red region of the spectrum is indicative of modification of the surface of the AuNP, resulting in a size increase which is reflected by increased absorption wavelength. The increase in particle size following addition of aptamers to the AuNP surface was also determined by DLS, yielding an average size increase (post-functionalisation) of 38.8 ± 5.1 nm (see Table S3 for complete DLS results). These results combined confirm the successful addition of aptamers to the particle surface.

### Aggregation assay - preliminary development

Two main aspects of the aggregation assay thought to improve assay performance are investigated during this study: i) the size of the AuNP core, and ii) the arrangement of the aptamers on the AuNP surface. The effect of AuNP core size on aggregation is initially investigated, in line with the findings of Krpetić et al. who showed that larger AuNP elicited an improved response in aggregation-based assays [26]. Here two sizes of AuNP are trialled; smaller ca. 15 nm AuNP, and larger ca. 50 nm AuNP. The aptamer-AuNP used for these experiments are functionalised in a single (rather than mixed) arrangement, yielding two AuNP solutions - AuNP-aptamer 1 and AuNP-aptamer 2. Equal volumes of the two functionalised AuNP solutions are then combined and mixed in a quartz cuvette (using a 1:1 ratio) prior to IL-6 addition and measurement (as detailed in the Methods section). Increasing volumes of IL-6 standard diluted in PBS, (10 mM phosphate buffer, 2.7 mM potassium chloride, 137 mM sodium chloride, pH 7.4) are then titrated into the solutions of aptamer-AuNP. After a 5 min incubation at room temperature, the test is complete and a UV-Vis absorption spectrum is taken after each addition of IL-6. Aggregation of the AuNP in the presence of IL-6 is indicated by a decrease in absorption intensity and a shift in the maximum wavelength.

**Fig. 2:**
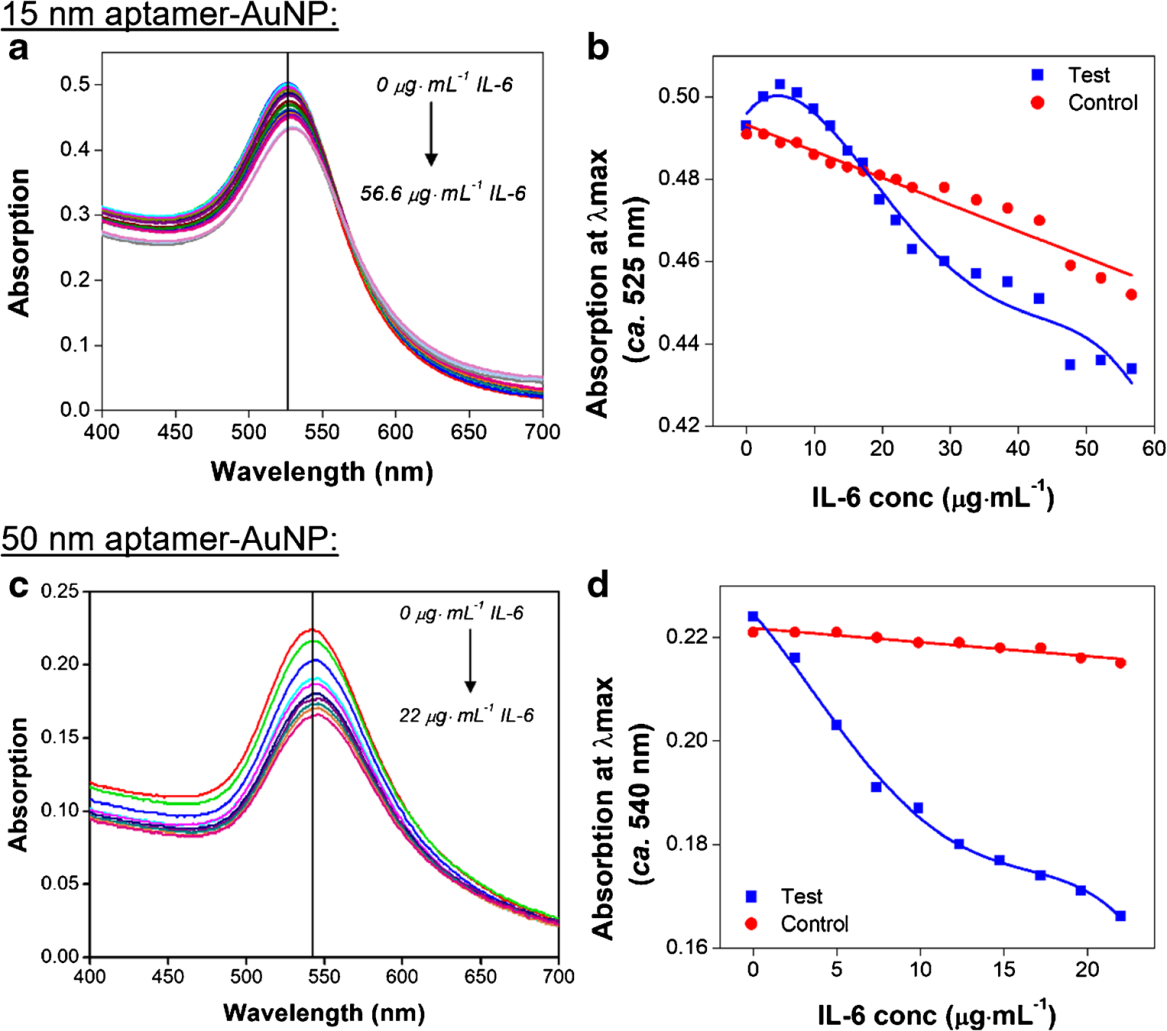
Comparing the effects of AuNP core size on IL-6 induced aggregation: 15 versus 50 nm (single) aptamer-AuNP. UV-Vis absorption spectra showing the decrease in the surface plasmon absorption band intensity in response to increasing concentrations of IL-6 for the 15 nm aptamer-AuNP (**a**) and for the 50 nm aptamer-AuNP (**c**). Aptamer-AuNP concentrations prior to IL-6 addition are an estimated 2.21 nM and 0.01 nM for the 15 nm and 50 nm aptamer-AuNP, respectively. Changes in the absorption intensity of 15 nm aptamer-AuNP (**b**, blue) and 50 nm aptamer-AuNP (**d**, blue) at the maximum wavelength with increasing concentrations of IL-6 are compared with those changes observed following addition of a buffer control (**b** and **d** red, respectively). Results shown are from a single set of experiments, representative of all repeats (*n* = 2)

Examples of decreasing peak absorption (of the aptamer-AuNP) in response to increasing concentrations of IL-6 from these initial tests are shown in Fig. 2a and c (for 15 and 50 nm aptamer-AuNP, respectively). It is hypothesised that, by using AuNP with a larger core size, the response to IL-6 in the μg·mL^−1^ range would be improved. The results show that for the smaller AuNP core-size (Fig. 2b), the response is not readily distinguishable from the control (in which equal volumes of buffer were added instead of IL-6). A shift in the wavelength maximum occurs in response to the addition of IL-6 (indicating enlarging of the particles, and therefore suggesting aggregation). For example, for the 15 nm AuNP core size, this shift is 4 nm after addition of IL-6 to 56.6 μg·mL^−1^ (compared to a 1.0 nm shift for the buffer control) in the experiment shown in Fig. 2. For the larger AuNP size, the IL-6 response (in terms of the decrease in absorption intensity) is noticeably different from the control (Fig. 2d) even at lower IL-6 concentrations (56.6 μg·mL^−1^ for 15 nm and 22 μg·mL^−1^ for 50 nm). It should be noted that a low concentration of 50 nm aptamer-AuNP (ca. 0.01 nM) is used in this experiment. A shift in the wavelength maximum of 4 nm (compared to a 1.5 nm shift for the buffer control) is again achieved in the experiment shown in Fig. 2. This time, half the concentration of IL-6 is used when compared with that used for the 15 nm AuNP core size. This response is also observed during repeat experiments (*n* = 2). It is therefore concluded that the 50 nm AuNP core size yielded an improved assay response in terms of particle aggregation when compared to smaller AuNP. Based on these results, and those of the previous set of experiments, 50 nm mixed aptamer-AuNP are used for all subsequent tests.

Experiments are also performed to determine whether AuNP functionalised with a single aptamer, or a mixture of both aptamers (in an approximate 1:1 ratio) would improve aggregation and test response. It is hypothesised that by co-functionalising the AuNP with both aptamers, this would improve the rate of aggregation by increasing the number of possible binding arrangements and preventing steric hindrance. For the co-functionalised AuNP, a 1:1 ratio of the two aptamers on the AuNP surface is assumed based on the equal amount of each aptamer applied during functionalisation (as detailed in the methods section). Complete characterisation of the resulting ratio was not performed as part of this pilot study. The titration of IL-6 was performed as described in the previous experiments.

**Fig. 3:**
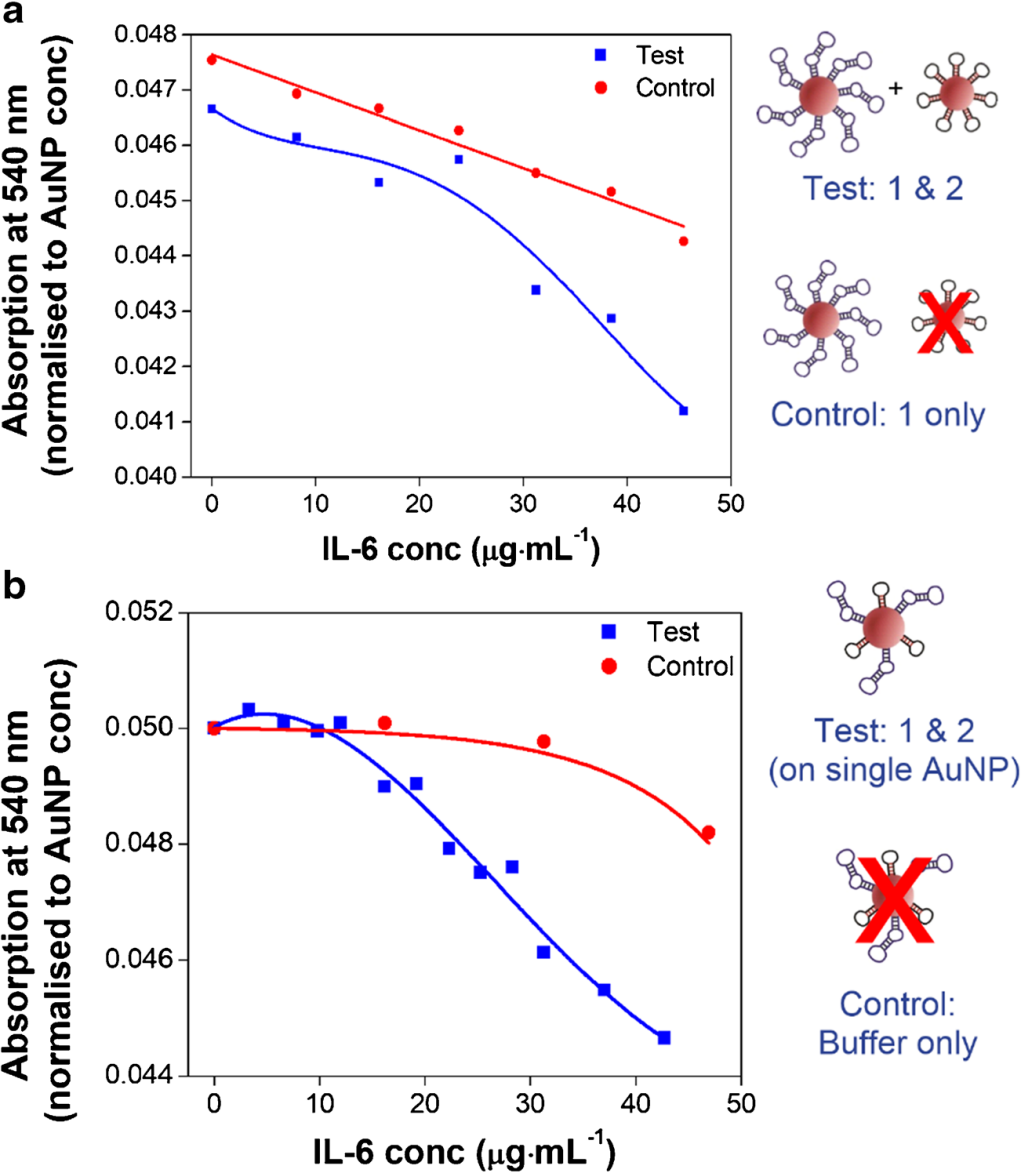
Comparing the effects of aptamer arrangement on IL-6-induced aggregation of ca. 50 nm AuNP: single aptamer versus mixed aptamer AuNP functionalisation. All results are expressed as absorption at 540 nm normalised to AuNP concentration, to correct for the difference in AuNP concentrations between the control and test in (**a**). Decreasing absorption intensity at the λ_max_ (ca. 540 nm) in response to increasing concentrations of IL-6 for the single-aptamer AuNP: A_max_ comparison with control (**a**). The final concentration of IL-6 in (**a**) is 45.45 μg·mL^−1^. The control used is a binding control, where IL-6 is titrated into a solution of single aptamer-AuNP (with the complimentary binding aptamer missing, as shown in the diagram). The response to increasing concentrations of IL-6 are also shown for the mixed-aptamer-AuNP: A_max_ comparison with buffer control (**b**). The final concentration of IL-6 in (**b**) is 42.7 μg·mL^−1^. Results shown are from a single set of experiments performed on ca. 50 nm AuNP, representative of all repeats (*n* = 2)

Results from an example experiment conducted using 50 nm-sized AuNP are shown in Fig. 3. Here the results shown are expressed as absorption at 540 nm normalised to AuNP concentration, to enable direct comparison with the control aptamer-AuNP 1 with the mixed aptamer-AuNP in Fig. 3a (the same units are used in Fig. 3b for consistency). For the single-aptamer-AuNP (Fig. 3a), the level of aggregation is not readily distinguishable from the binding control (in which only one of the two aptamer-AuNP required for aggregation was present). However, for the mixed-aptamer-AuNP, the IL-6 response is markedly different from the buffer control (Fig. 3b). The mixed-aptamer-AuNP therefore yields more aggregation when compared to the single-aptamer-AuNP, suggesting that a greater number of binding opportunities do improve the response of the test. This improved response is also observed during repeat experiments using ca. 50 nm-sized AuNP (*n* = 2). Similar experiments are performed using ca. 15 nm sized AuNP. The effects of co-functionalisation on AuNP of this size is less obvious than with the larger sized AuNP due to the lower degree of aggregation achieved (as previously discussed). Based on the results exemplified in Fig. 3, 50 nm mixed-aptamer-AuNP are used for all subsequent experiments. The investigation of other co-functionalisation ratios were not explored in this pilot study. However, in future, these may be considered as a means of optimising assay sensitivity for biological application.

### Aggregation assay results – Analysing IL-6 standard solutions

The performance of the assay, now comprising of 50 nm mixed-aptamer-AuNP, is further determined via titrations of increasing concentrations of IL-6 (in buffer). This time a larger IL-6 concentration range than previously attempted (to a final concentration of 109.4 μg·mL^−1^) is explored. The starting concentration of the 50 nm mixed-aptamer AuNP (prior to addition of IL-6) is also increased 5-fold, from ca. 0.01 nM (as used in previous experiments, e.g. as shown in Fig. 3b), to ca. 0.05 nM. This change is made with the aim of producing a visual colour change.

**Fig. 4:**
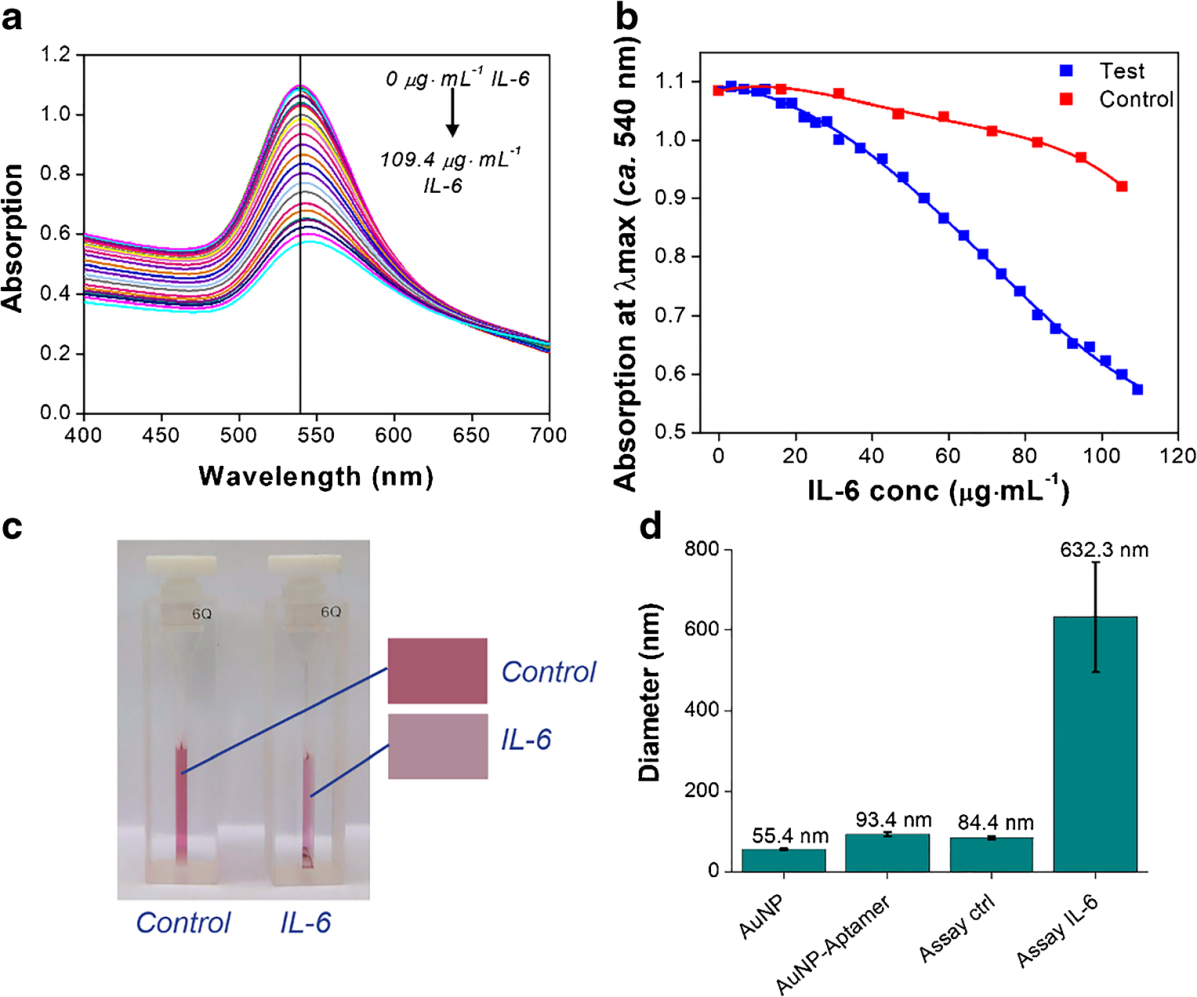
Aggregation assay (comprising of 50 nm mixed-aptamer-AuNP) results for IL-6 standard in buffer. Decreasing absorption intensity at the λ_max_ (ca. 540 nm) in response to increasing concentrations of IL-6: UV-Vis absorption spectra (**a**), and absorption at λ_max_ comparison with buffer control (**b**). The visible colour change of the final test solution (containing 109.4 μg·mL^−1^ IL-6) compared with the control sample containing an equivalent volume of blank buffer (**c**). Results shown in **a**, **b** and **c** are from a single experiment, representative of all repeats (*n* = 2). Aptamer-AuNP concentration prior to IL-6 addition is estimated at 0.05 nM. Characterisation of the cAuNP core, aptamer-AuNP, test control, and the test solution (containing 109.4 μg·mL^−1^ IL-6) by DLS (**d**). The size estimates shown are based on 10 repeat measurements (n = 2 separate experiments); error bars denote SD of repeat measurements

Results from a typical experiment are shown in Fig. 4a, b and c. The UV-Vis absorption spectrum shows a steady decrease in the intensity of the surface plasmon absorption band at ca. 540 nm in response to increasing IL-6 (Fig. 4a). A typical red shift in the absorption wavelength maximum of 7 nm is observed in the test solution in this experiment (compared to a zero nm shift in the control solution). This shift further confirms the aggregation of the aptamer-AuNP in response to IL-6. A comparison between absorption at the λ_max_ (ca. 540 nm) in response to either IL-6 or an equal volume of blank buffer is shown in Fig. 4b. There is a clear response to increasing IL-6 addition which is readily distinguishable from the dilution effect illustrated by the control sample. The aggregation of the aptamer-AuNP is further demonstrated in Fig. 4c, which shows the colour change in response to 109.4 μg·mL^−1^ IL-6 (compared with the control). Characterisation of the AuNP core, aptamer-functionalised AuNP, and control versus IL-6 aggregation test samples was also performed by DLS. The DLS results (Fig. 4d) indicate an original hydrodynamic diameter of the AuNP of approximately 50 nm, which increases by an estimated 30–40 nm with the addition of aptamers to the AuNP surface. Results shown in Fig. 4d are cumulative results from two repeat experiments (see Table S3 for complete DLS results). In the absence of IL-6, the control sample (in which equal volumes of buffer are added instead of IL-6) results in a size estimate comparable to that of the aptamer-functionalised AuNP, confirming a lack of aggregation. Conversely, in the presence of IL-6, large AuNP aggregates estimated at 600 nm in size are formed, confirming successful aggregation of the aptamer-AuNP in the presence of the marker of inflammation.

**Fig. 5:**
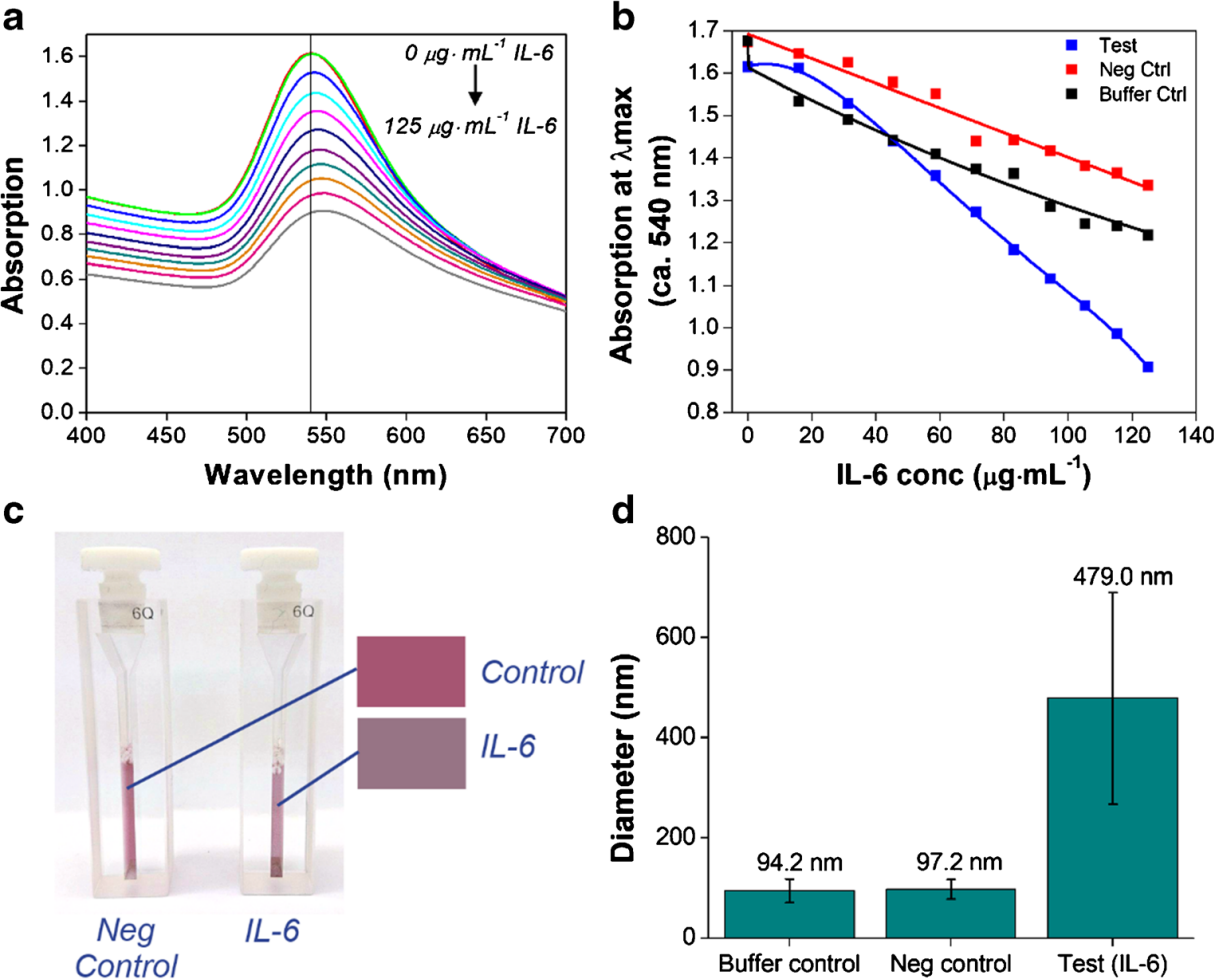
Aggregation assay (comprising of 50 nm mixed-aptamer-AuNP) results for IL-6 standard in a broadly representative sample matrix. Decreasing absorption intensity at the λ_max_ (ca. 540 nm) in response to increasing concentrations of IL-6: UV-Vis absorption spectra (**a**), and changes in the absorption maxima in comparison with controls (**b**). The ‘buffer control’ contains HSA only; the ‘negative control’ contains both HSA and a mixture of human cytokines and chemokines as a representative sample matrix (as listed in Table S2). The visible colour change of the final test solution (containing 125 μg·mL^−1^ IL-6), compared with the ‘negative control’ (**c**). Results shown in **a**, **b** and **c** are from a single experiment, representative of all repeats (*n* = 2). Aptamer-AuNP concentration prior to IL-6 addition is estimated at 0.08 nM. Characterisation of the controls and the test solution (containing 125 μg·mL^−1^ IL-6) by DLS (**d**). The size estimates are based on 10 repeat measurements (n = 2 separate experiments); error bars denote SD of repeat measurements

The performance of the aggregation assay is further determined by another titration of increasing volumes of IL-6 (to a final concentration of 125 μg·mL^−1^), but this time in a mixed protein solution broadly representative of a biological matrix. The mixed protein solution comprises of human serum albumin (HSA) and 34 arbitrary human cytokines and chemokines, notably including human IL-6 (see Table S2 for detailed composition of the matrix). Results from a typical experiment are shown in Fig. 5a, b and c. The UV-Vis absorption spectrum, showing a steadily decreasing absorption intensity at the λ_max_ (ca. 540 nm) in response to increasing IL-6 (comparable to that obtained using IL-6 in buffer, Fig. 5a). A red-shift in the λ_max_ of 8.5 nm is again observed in this representative experiment (compared to 1.0 ± 0.5 nm for the two controls). The observed shift confirms aggregation of the aptamer-AuNP in response to IL-6 without interference from the sample matrix. A comparison between absorption at the λ_max_ (ca. 540 nm) in response to either IL-6 or an equal volume of two control buffers is shown in Fig. 5b. Here the controls consist of a buffer containing HSA only (‘buffer control’), and a buffer containing both HSA and the human protein mix (‘negative control’). The aggregation of the aptamer-AuNP in response to IL-6 is again clearly distinguishable from the dilution effect seen with both buffers. By separating the response of the ‘negative control’ from the ‘buffer’ (dilution effect) control, this result suggests that no obvious cross-reactivity with human IL-6 (when present at low concentrations) has occurred during this preliminary test. The aggregation of the aptamer-AuNP is further demonstrated in Fig. 5c, which shows the colour change in response to IL-6 versus the ‘negative control’. The ‘buffer control’ also does not yield a colour change (as shown in Fig. S2). Characterisation of both controls and the IL-6 aggregation test sample was again performed by DLS. The DLS results (Fig. 5d) show that both controls result in a size estimate comparable to that of the aptamer-functionalised AuNP (see Fig. 4d), confirming a lack of aggregation. Conversely, in the presence of IL-6, large AuNP aggregates of around 500 nm in size are formed, confirming successful aggregation of the aptamer-AuNP.

In this pilot study we have demonstrated the application of this preliminary assay format for the detection of IL-6 in the low (1–125) μg·mL^−1^ range. In biological samples such as mouse plasma, IL-6 can be present at concentrations between 1 and 1500 pg·mL^−1^ (depending on the health of the animal) [27]. Concentrations of IL-6 in human serum are in the low pg·mL^−1^ range in healthy controls and increase significantly in various disease states. For example, Robak et al. reported serum IL-6 concentrations of 0.5–16.6 pg·mL^−1^ in healthy controls and 1.5–234 pg·mL^−1^ in rheumatoid arthritis patients [28]. In intensive care unit patients with sepsis, Damas et al. reported IL-6 serum concentrations of 200–461,850 pg·mL^−1^ [29]. Full optimisation of the present assay to achieve a lower limit of detection, and a comparison with current state-of-the-art point-of-care assays is necessary, but was beyond the scope of this small pilot study. There is considerable opportunity for further development and optimisation to achieve a lower limit of detection, for example by adapting the concentration of aptamer-AuNP in the test solution, or by investigating different co-functionalisation ratios for improved binding efficacy. With further optimisation, a test of this nature may be readily applied for the rapid analysis of relevant biological samples. Here we have demonstrated the potential of this assay format, with a view to future clinical application. This will involve development and complete validation of this promising assay format with appropriate aptamers targeted to human inflammatory markers.

## Conclusions

A proof-of-concept aptamer-based assay format for detecting IL-6, a key marker of acute inflammation, has been presented. The assay is based on the aggregation of AuNP coated in two complimentary “sandwich-style” aptamers, which each bind to different sites on the IL-6 peptide. The aggregation of AuNP in the presence of IL-6 induces a visible colour change in just 5 min, with a corresponding change in absorption intensity at ca. 540 nm which can be monitored by spectrophotometer or plate reader. Size increase upon aggregation can be also followed by DLS measurements. In this pilot study, key aspects of the assay format (AuNP size and aptamer arrangement) are investigated to improve the level of aggregation and sensitivity achieved. The assay is successfully applied to the detection of murine IL-6 (in the μg·mL^−1^ range) in buffer, and in a mixed protein solution broadly representative of a biological sample matrix. This proof-of-concept study does not, as yet, possess the sensitivity to detect the low levels of IL-6 in healthy serum/plasma. More work is needed to improve the sensitivity. During infections and sepsis very high levels are known to occur in patients (see above). With further development and optimisation to improve the limit of detection, this assay format may enable rapid and straightforward detection of acute inflammation in either a mouse model, or in mouse-derived cell culture supernatant. This pilot study was performed as a proof-of-concept demonstration of aptamer-functionalised AuNP, with a view to developing a similar assay targeting human inflammatory markers for analysing patient samples in future. The principle of our assay may, with further development, lead to a rapid point-of-care test in patients.

## Electronic supplementary material

ESM 1 (PDF 758 kb)

## References

[1] Hunter CA, Jones SA (2015). IL-6 as a keystone cytokine in health and disease. Nat Immunol.

[2] Kumar RG, Boles JA, Wagner AK (2015). Chronic inflammation after severe traumatic brain injury: characterization and associations with outcome at 6 and 12 months postinjury. J Head Trauma Rehabil.

[3] Nwachuku EL, Puccio AM, Adeboye A, Chang Y-F, Kim J, Okonkwo DO (2016). Time course of cerebrospinal fluid inflammatory biomarkers and relationship to 6-month neurologic outcome in adult severe traumatic brain injury. Clin Neurol Neurosurg.

[4] Wu W, Guan Y, Zhao G, Fu X-J, Guo T-Z, Liu Y-T, Ren X-L, Wang W, Liu H-R, Li Y-Q (2016). Elevated IL-6 and TNF-α levels in cerebrospinal fluid of subarachnoid hemorrhage patients. Mol Neurobiol.

[5] Lenski M, Huge V, Briegel J, Tonn J-C, Schichor C, Thon N (2017). Interleukin 6 in the cerebrospinal fluid as a biomarker for onset of vasospasm and ventriculitis after severe subarachnoid hemorrhage. World Neurosurg.

[6] Takahashi W, Nakada T-A, Abe R, Tanaka K, Matsumura Y, Oda S (2014). Usefulness of interleukin 6 levels in the cerebrospinal fluid for the diagnosis of bacterial meningitis. J Crit Care.

[7] Prasad R, Kapoor R, Srivastava R, Mishra OP, Singh TB (2014). Cerebrospinal fluid TNF-α, IL-6, and IL-8 in children with bacterial meningitis. Pediatr Neurol.

[8] Bloos F, Reinhart K (2014). Rapid diagnosis of sepsis. Virulence.

[9] Bayer O, Schwarzkopf D, Stumme C, Stacke A, Hartog CS, Hohenstein C, Kabisch B, Reichel J, Reinhart K, Winning J (2015). An early warning scoring system to identify septic patients in the prehospital setting: the PRESEP score. Acad Emerg Med.

[10] Molano Franco D, Arevalo-Rodriguez I, Roqué i Figuls M, Zamora J (2015). Interleukin-6 for diagnosis of sepsis in critically ill adult patients. Cochrane Database Syst Rev.

[11] Mancini N, Carletti S, Ghidoli N, Cichero P, Burioni R, Clementi M (2010). The era of molecular and other non-culture-based methods in diagnosis of sepsis. Clin Microbiol Rev.

[12] Hack CE, De Groot ER, Felt-Bersma R, Nuijens JH, Van Schijndel RS, Eerenberg-Belmer A, Thijs LG, Aarden LA (1989). Increased plasma levels of interleukin-6 in sepsis. Blood.

[13] Chaemsaithong P, Romero R, Korzeniewski SJ, Martinez-Varea A, Dong Z, Yoon BH, Hassan SS, Chaiworapongsa T, Yeo L (2016). A rapid interleukin-6 bedside test for the identification of intra-amniotic inflammation in preterm labor with intact membranes. J Matern Fetal Neonatal Med.

[14] Sefah K, Shangguan D, Xiong X, O'donoghue MB, Tan W (2010). Development of DNA aptamers using cell-SELEX. Nat Protoc.

[15] Gopinath SC, Lakshmipriya T, Chen Y, Phang W-M, Hashim U (2016). Aptamer-based 'point-of-care testing'. Biotechnol Adv.

[16] Toh SY, Citartan M, Gopinath SC, Tang T-H (2015). Aptamers as a replacement for antibodies in enzyme-linked immunosorbent assay. Biosens Bioelectron.

[17] Marín MJ, Rashid A, Rejzek M, Fairhurst SA, Wharton SA, Martin SR, McCauley JW, Wileman T, Field RA, Russell DA (2013). Glyconanoparticles for the plasmonic detection and discrimination between human and avian influenza virus. Org Biomol Chem.

[18] Pavlov V, Xiao Y, Shlyahovsky B, Willner I (2004). Aptamer-functionalized au nanoparticles for the amplified optical detection of thrombin. J Am Chem Soc.

[19] Marín MJ, Schofield CL, Field RA, Russell DA (2015). Glyconanoparticles for colorimetric bioassays. Analyst.

[20] Sabela M, Balme S, Bechelany M, Janot JM, Bisetty K (2017). A review of gold and silver nanoparticle-based colorimetric sensing assays. Adv Eng Mater.

[21] Giorgi-Coll S, Blunt-Foley H, Hutchinson PJ, Carpenter KL (2017). Heparin-gold nanoparticles for enhanced microdialysis sampling. Anal Bioanal Chem.

[22] Turkevich J, Stevenson PC, Hillier J (1953). The formation of colloidal gold. J Phys Chem.

[23] Bastús NG, Comenge J, Puntes V (2011). Kinetically controlled seeded growth synthesis of citrate-stabilized gold nanoparticles of up to 200 nm: size focusing versus ostwald ripening. Langmuir.

[24] Zhang X, Servos MR, Liu J (2012). Instantaneous and quantitative functionalization of gold nanoparticles with thiolated DNA using a pH-assisted and surfactant-free route. J Am Chem Soc.

[25] Marín MJ, Rackham BD, Round AN, Howell LA, Russell DA, Searcey M (2013). A rapid screen for molecules that form duplex to duplex crosslinks in DNA. Chem Commun.

[26] Krpetić Ž, Guerrini L, Larmour IA, Reglinski J, Faulds K, Graham D (2012). Importance of nanoparticle size in colorimetric and SERS-based multimodal trace detection of Ni(II) ions with functional gold nanoparticles. Small.

[27] Nukina H, Sudo N, Aiba Y, Oyama N, Koga Y, Kubo C (2001). Restraint stress elevates the plasma interleukin-6 levels in germ-free mice. J Neuroimmunol.

[28] Robak T., Gladalska A., Stepień H., Robak E. (1998). Serum levels of interleukin-6 type cytokines and soluble interleukin-6 receptor in patients with rheumatoid arthritis. Mediators of Inflammation.

[29] Damas P, Ledoux D, Nys M, Vrindts Y, De Groote D, Franchimont P, Lamy M (1992). Cytokine serum level during severe sepsis in human IL-6 as a marker of severity. Ann Surg.

