# Supplementary material for: Aptamer-modified gold nanoparticles for rapid aggregation-based detection of inflammation: an optical assay for interleukin-6
Source: Mikrochim Acta. 2019 Dec 4;187(1):13. doi: 10.1007/s00604-019-3975-7 (PMC6892788; doi:10.1007/s00604-019-3975-7)
Supplement: Supplementary file 1 — (PDF 758 kb) [file 604_2019_3975_MOESM1_ESM.pdf]

Electronic Supporting Material
on the Microchimica Acta publication entitled

Aptamer-modified gold nanoparticles for rapid aggregation-based detection of inflammation: an optical assay for interleukin-6

Susan Giorgi-Coll[1]*, María J. Marín[2]*, Olajumoke Sule[3], Peter J. Hutchinson[1,4], Keri L.H. Carpenter[1,4].

1. **Division of Neurosurgery, Department of Clinical Neurosciences, University of Cambridge, Box 167, Cambridge Biomedical Campus, Cambridge, CB2 0QQ, UK**
2. **School of Chemistry, University of East Anglia, Norwich Research Park, Norwich, NR4 7TJ, UK**
3. **Clinical Microbiology and Public Health Laboratory, Cambridge University Hospitals NHS Trust, Box 236, Addenbrooke's Hospital, Cambridge, CB2 0QQ, UK**
4. **Wolfson Brain Imaging Centre, Department of Clinical Neurosciences, University of Cambridge, Box 65, Cambridge Biomedical Campus, Cambridge, CB2 0QQ, UK**

**Corresponding authors:*

Introduction

Table S1 An overview on recently reported methods, including nanomaterial-based optical methods, for the determination of IL-6

Recognition Element	Method Applied	Linear Ranges ($pg\cdot mL^{-1}$)	Detection Limit ($pg\cdot mL^{-1}$)	Reference
Antibody	Photoelectrochemical immunoassay	$0.1 – 1.0 \times 10^{5}$	0.033	[S1]
Antibody	Electrochemical immunoassay	$1.0 \times 10^{-3} – 9.0 \times 10^{3}$	3.2×10^{-4}	[S2]
Antibody	Electrochemiluminescent immunoassay	$1.0 \times 10^{-5} – 9.0 \times 10^{3}$	3.5×10^{-6}	[S2]
Antibody	Fluorescence	0.4 – 400	0.1	[S3]
Antibody	Electrochemiluminescent immunoassay	$2.0 – 2.0 \times 10^{4}$	0.65	[S4]
Antibody	Photoelectrochemical immunoassay	$1.0 \times 10^{-6} – 10.0$	0.737×10^{-6}	[S5]
Antibody	Magnetic Colorimetric Immunoassay	$0.1 – 1.0 \times 10^{3}$	0.04	[S6]
Antibody	Immunochromatrographic Strip	$62.5 \times 10^{3} – 2.0 \times 10^{6}$	62.5×10^{3}	[S7]
Antibody	Liquid-gated field effect transistor	4.7 – 300	1.53	[S8]
Antibody	Photoelectrochemical immunoassay	$1.0 – 1.0 \times 10^{5}$	0.38	[S9]
Antibody	Electrochemical immunoassay	$1.0 \times 10^{-5} – 100$	1.0×10^{-5}	[S10]
Aptamer	Colorimetric	$3.3 \times 10^{3} – 125 \times 10^{3}$	1.95×10^{3}	This work

Experimental

AuNP synthesis and characterisation

Citrate-stabilised gold nanoparticles (cAuNP) of *ca.* 15 nm were synthesised according to the method of Turkevich *et al.* [S11]. Briefly, aqueous solutions of $HAuCl_4\cdot 3H_2O$ (12.5 mg, 32 µmol, in 100 mL water) and sodium citrate tribasic dihydrate (50 mg, 168 µmol, in 50 mL water) were prepared and heated to 60 °C. The two solutions were rapidly combined while stirring vigorously. The temperature was increased to 85 °C and the solution was stirred for 2.5 h, resulting in a ruby-red gold nanoparticle solution. After cooling to room temperature, the solution was filtered through a Millipore syringe filter unit (0.45 µm). Characterisation was performed by UV-Vis as described in the Materials and Equipment section. The particle concentration in the citrate-stabilised gold nanoparticles solution was approximately 3 nM [S11].

Synthesis of *ca.* 50 nm cAuNP was performed using the seeded growth method (for AuNPs of up to 180 nm) of Bastús *et al.* [S12]. AuNP seeds (*ca.* 10 nm) were first prepared by boiling an aqueous solution of sodium citrate (150 mL, 2.2 mM) in a three-necked round bottom flask under reflux. Once boiling, 1 mL of a 25 mM $HAuCl_4{\cdot}3H_2O$ solution (at room temperature) was rapidly injected under vigorous stirring. A colour change from pale yellow to bluish grey and finally a soft pink occurred within 10 min, signalling the formation of the cAuNP seeds. Larger cAuNP synthesis was then performed immediately and in the same reaction vessel. The soft pink solution was first cooled to 90 °C. Then, 1 mL of a $HAuCl_4{\cdot}3H_2O$ solution (25 mM, at room temperature) was injected under vigorous stirring. After 30 min, the reaction was finished, as indicated by a colour change from soft pink to ruby red. This process was repeated a further two times. Afterward, a dilution step was performed by extracting 55 mL of solution, and subsequently adding 53 mL of dH_2O and 2 mL of aqeous sodium citrate (60 mM) (both at room temperature). All steps were performed at 90 °C under vigorous stirring. This diluted solution was then used as a seed solution, and the entire process was repeated again as follows. After a short incubation (5 min or less) to allow the added solutions to equilibrate to 90 °C, 1 mL of aqueous $HAuCl_4{\cdot}3H_2O$ (25 mM, at room temperature) was injected and the solution was stirred vigorously (at 90 °C) for 30 min. This process was repeated a further two times, after which the dilution step was performed as described previously. A single 'growth step' comprises of this entire sequence (3x 1 mL of 25 mM $HAuCl_4{\cdot}3H_2O$ with 30 min incubation between, followed by the dilution step consisting of extraction of 55 mL of solution, and addition of 53 mL + 2 mL of additional solutions). A total of six growth steps were performed in order to obtain *ca.* 50 nm cAuNP. The extracted solutions obtained during the dilution steps, and the final solution of cAuNP were rapidly cooled to room temperature (on ice) to stop the reaction. After cooling, the solution was filtered through a Miller GP syringe driven filter unit (0.45 µm). Characterisation of the extracted and final solutions were performed by UV-Vis as described in the Materials and Equipment section, enabling the growth of the AuNP to be monitored. The particle concentration in the final *ca.* 50 nm cAuNP solution was approximately 0.2 nM [S12].

TEM was used to establish the size and morphology of the *ca.* 15 and 50 nm cAuNP. 10 µL of the cAuNP in solution was placed onto a holey carbon film 300 mesh copper grid. Excess solution was removed from the sample by touching the side of the grid with adsorbent tissue paper. The grid was dried overnight at room temperature before analysis by TEM, as previously described. Size measurement of the cAuNP was performed using ImageJ software. DLS size measurements of the 15 nm cAuNP were performed on a Malvern Zetasizer, as described previously. Size measurements were recorded at 25 °C, with a backscattering and forward scattering angles of detection (173° and 13°). Water was selected as the dispersant

with viscosity and refractive index (RI) of 0.8872 cP and 1.330 cP, respectively. Measurements of the 50 nm cAuNP were performed in a DynoPro (DLS) Plate Reader II using Corning 96-well plastic-bottom plates (200 µL of sample/well), at 24 °C and with a 100 sec acquisition time (3 reads/well). All measurements were performed in duplicate.

Table S2 Composition of the 'representative biological matrix' - concentrations of the human cytokine and chemokine standards present in the mixed protein stock solution (in Universal Assay Buffer with 0.5 mg·mL^{-1} HSA)

Cytokine/Chemokine	Concentration (pg·mL^{-1})*
Eotaxin	25
GM-CSF	543
GRO-alpha	95
IFN-alpha	22.5
IFN-gamma	306
IL-1alpha	22
IL-1beta	84
IL-1ra	1438
IL-2	175
IL-4	463
IL-5	263
IL-6	334
IL-7	20
IL-8	89
IL-9	315
IL-10	92
IL-12p70	262
IL-13	88
IL-15	125
IL-17alpha	74
IL-18	280
IL-21	319
IL-22	1082
IL-23	559
IL-27	1002
IL-31	546
IP-10	88
MCP-1	65
MIP-1alpha	64
MIP-1beta	135
RANTES	36
SDF-1alpha	398
TNF-alpha	267
TNF-beta	238

Results and Discussion

AuNP synthesis and characterisation

TEM is used to visualise the 15 and 50 nm cAuNP (shown in **Fig. S1a** and **d**, respectively). The average diameters of the cAuNP, measured from the TEM images, are 15.0 ± 1.3 nm and 53.0 ± 5.2 nm (n = 100) (**Fig. S1b** and **e)**. DLS is also used to estimate the size of the cAuNP. The average sizes of the cAuNP, estimated by DLS, are 16.5 ± 0.21 nm and 55.4 ± 1.9 nm (based on 3 repeat measurements per sample). cAuNP absorb light in the visible spectrum with a maximum absorption intensity and wavelength that are size-dependent. For the 15 nm cAuNP, the surface plasmon absorption band exhibits a maximum at *ca.* 520 nm, yielding a spectrum as shown in **Fig. S1c**. For the 50 nm cAuNP, the surface plasmon absorption band exhibits a maximum at approximately 540 nm, as demonstrated in **Fig. S1f**. The binding of the aptamers to the surface of the AuNP is demonstrated by a shift in the absorption wavelength of approximately 3-4 nm following functionalisation, as shown in **Fig. S1c** and **f** for 15 nm and 50 nm AuNPs, respectively. This shift to the red region of the spectrum is indicative of modification of the surface of the AuNP, resulting in a size increase which is reflected by increased absorption wavelength. The increase in particle size following addition of aptamers to the AuNP surface is also determined by DLS, yielding an average size increase (post-functionalisation) of 38.8 ± 5.1 nm (see **Table S3** for complete DLS results). These results combined confirm the successful addition of aptamers to the particle surface.

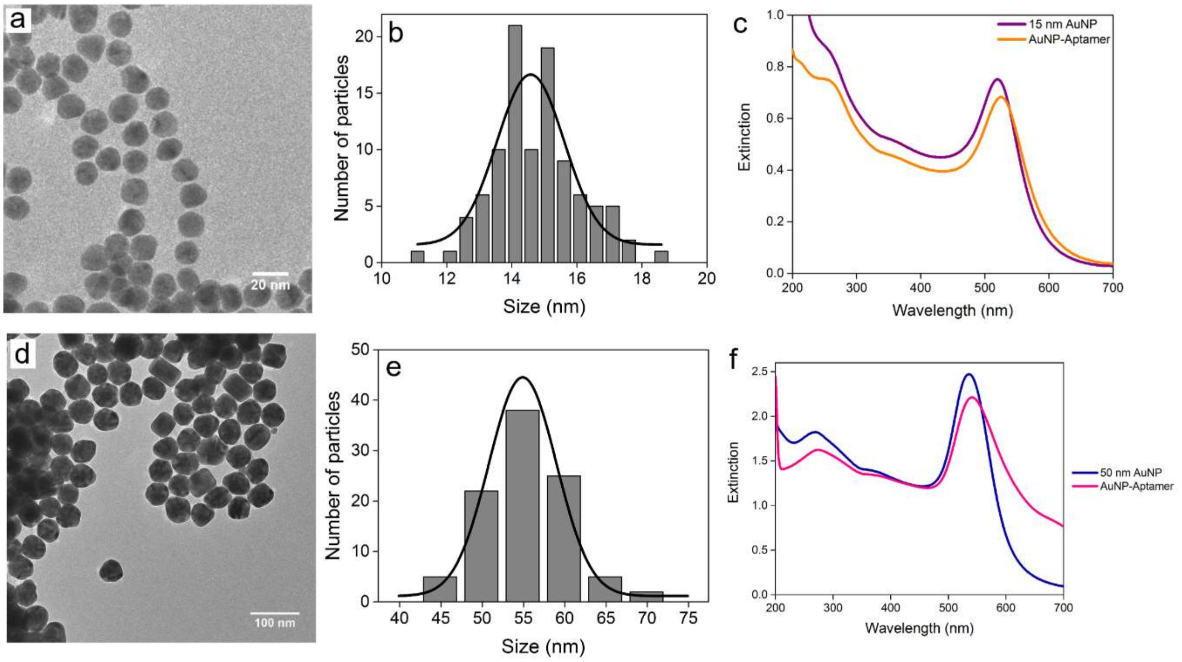

Fig. S1 Characterisation of the aptamer-AuNP by TEM and UV-Vis spectrophotometry. TEM images of the 15 and 50 nm citrate AuNP are shown in (**a**) and (**d**), respectively. The average sizes of the cAuNP, estimated from the TEM images (n = 100), were 15.0 ± 1.3 nm (**b**) and 53.0 ± 5.2 nm (**e**). UV-Vis absorption spectra of the 15 and 50 nm cAuNP and aptamer-AuNP are shown in (**c**) and (**f**), respectively. Addition of aptamers to the AuNP surface led to a red shift in the absorption maxima (at *ca.* 520 nm for the 15 nm AuNP, and *ca.* 540 nm for the 50 nm AuNP) of 3.5 ± 1.0 nm (based on 3 repeat measurements/AuNP size)

Table S3 The particle size of: *ca.* 15 and 50 nm cAuNP, aptamer-functionalised AuNP, and aggregation assay solutions (as determined by DLS)

**Sample**	**Average Diameter ± SD (nm)**
ca. 15 nm cAuNP	16.50 ± 0.11
ca. 15 nm aptamer-AuNP	55.96 ± 5.30
ca. 50 nm cAuNP	54.58 ± 1.00
ca. 50 nm aptamer-AuNP	93.40 ± 5.07*
ca. 50 nm mixed aptamer-AuNP: buffer test control (without IL-6)	84.43 ± 3.92*
ca. 50 nm mixed aptamer-AuNP: buffer test (with 109.4 μg·mL^{-1} IL-6)	632.29 ± 137.62
ca. 50 nm mixed aptamer-AuNP: "representative sample" buffer control (without IL-6)	94.17 ± 23.18
ca. 50 nm mixed aptamer-AuNP: "representative sample" negative control (without IL-6)	97.18 ± 19.58
ca. 50 nm mixed aptamer-AuNP: "representative sample" test (with 125 μg·mL^{-1} IL-6)	479.02 ± 211.92*

SD: Standard Deviation (n = 3) or *(n = 4)

Aggregation assay results – detecting IL-6 standard solutions

The aggregation assay is performed titrating increasing volumes of IL-6 in a mixed protein solution broadly representative of a biological matrix (see **Table S2** for detailed composition of the matrix – components include human serum albumin and 36 arbitrary human cytokines and chemokines). A decrease in the absorption intensity at the λ_{max} (*ca.* 540 nm) and a red shift in the absorption maximum are observed in response to increasing IL-6 concentration (from 0 to 125 $\mu g \cdot mL^{-1}$), confirming the aggregation of the aptamer-AuNP. Two buffers are used as control: 1) a buffer containing HSA only ('buffer control'), and 2) a buffer containing both HSA and the human protein mix ('negative control'). No changes in the absorption intensity at the λ_{max} (*ca.* 540 nm) are observed when equal volumes of the controls are added to the aptamer-AuNP. The aggregation of the particles in the presence of IL-6 in the mixed protein matrix is confirmed by a visual colour change (**Fig. S2 – right**) that is not observed when the particles were treated with the 'buffer control' (**Fig. S2 – left**).

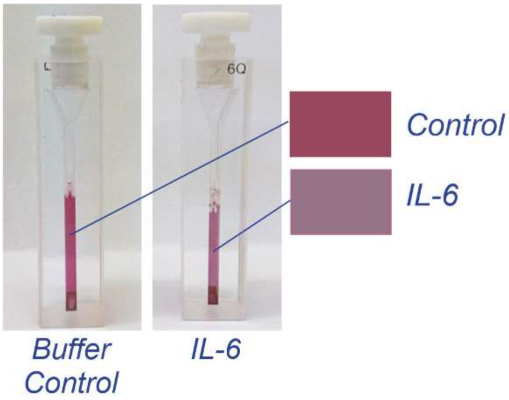

Fig. S2 The visible colour change of the final test solution (containing 125 $\mu g \cdot mL^{-1}$ IL-6), compared with the 'buffer control' (containing 0.5 $mg \cdot mL^{-1}$ HSA only)

References

[S1] Liu XP, Xie XL, Wei YP, Mao CJ, Chen JS, Niu HL, Song JM, Jin BK (2018). Photoelectrochemical immunoassay for human interleukin 6 based on the use of perovskite-type $LaFeO_3$ nanoparticles on fluorine-doped tin oxide glass. Microchimica Acta 185:52

[S2] Liu N, Yi H, Lin Y, Zheng H, Zheng X, Lin D, Dai H (2018). Combined electrochemiluminescent and electrochemical immunoassay for interleukin 6 based on the use of TiO_2 mesocrystal nanoarchitectures. Microchimica Acta, 185:277

[S3] Zhang KX, Liu GZ, Goldys EM (2018) Robust immunosensing system based on biotin-streptavidin coupling for spatially localized femtogram mL^{-1} level detection of interleukin-6. Biosens Bioelectron 102:80–86

[S4] Yang Y, Liu Q, Liu XP, Liu PZ, Mao CJ, Niu HL, Jin BK, Zhang SY (2016) Multifunctional reduced graphene oxide (RGO)/Fe_3O_4/CdSe nanocomposite for electrochemiluminescence immunosensor. Electrochim Acta 190:948-955

[S5] Gong LS, Dai H, Zhang SP, Lin YY (2016) Silver iodide-chitosan nanotag induced biocatalytic precipitation for self-enhanced ultrasensitive photocathodic immunosensor. Anal Chem 88:5775–5782

[S6] Peng J, Guan JF, Yao HQ, Jin XY (2016) Magnetic colorimetric immunoassay for human interleukin-6 based on the oxidase activity of ceria spheres. Anal Biochem 492:63–68

[S7] Man Y, Lv XF, Iqbal J, Peng G, Song D, Zhang CX, Deng YL (2015) Microchip based and immunochromatographic strip assays for the visual detection of interleukin-6 and of tumor necrosis factor α using gold nanoparticles as labels. Microchim Acta 182:597–604

[S8] Huang J, Chen H, Niu W, Fam DWH, Palaniappan A, Larisika M, Faulkner SH, Nowak C, Nimmo MA, Liedberg B, Tok AIY (2015) Highly manufacturable graphene oxide biosensor for sensitive Interleukin-6 detection. RSC Adv 5:39245-39251

[S9] Fan GC, Ren XL, Zhu C, Zhang JR, Zhu JJ (2014) A new signal amplification strategy of photoelectrochemical immunoassay for highly sensitive interleukin-6 detection based on TiO_2/CdS/CdSe dual co-sensitized structure. Biosens Bioelectron 59:45-53.

[S10] Yang T,Wang S, Jin HL, BaoWW, HuangSM,Wang JC (2013) An electrochemical impedance sensor for the label-free ultrasensitive detection of interleukin-6 antigen. Sensors Actuators B 178:310-315

[S11] Turkevich J, Stevenson PC, Hillier J (1953) The formation of colloidal gold. J Phys Chem 57:670-673

[S12] Bastús NG, Comenge J, Puntes V (2011) Kinetically controlled seeded growth synthesis of citrate-stabilized gold nanoparticles of up to 200 nm: size focusing versus ostwald ripening. Langmuir 27:11098-11105
